# Comparative Analysis between the Gail, Tyrer-Cuzick and BRCAPRO Models for Breast Cancer Screening in Brazilian Population

**DOI:** 10.31557/APJCP.2019.20.11.3407

**Published:** 2019

**Authors:** Kely Paviani Stevanato, Raíssa Bocchi Pedroso, Pedro Iora, Lander dos Santos, Fernando Castilho Pelloso, Willian Augusto de Melo, Maria Dalva de Barros Carvalho, Sandra Marisa Pelloso

**Affiliations:** 1 *Master's Degree in Health Sciences of the Graduate Program in Health Sciences, *; 2 *Postdoctoral fellowship of the Postgraduate Program in Health Sciences, *; 3 *Student of the Medical School, *; 6 *Lecturer at the Department of Medicine at Unicesumar University and Professor at the Department of Postgraduate Science in Health, State University of Maringá (UEM), *; 4 *Student of the Medical at Federal University of Paraná (UFPR), *; 5 *Lecturer at the Nursing Department of the Paraná State University, Paranavaí campus Brazil. *

**Keywords:** Breast cancer, screening, correlation between predictive risk models

## Abstract

**Objective:**

To analyze the diagnostic accuracy of predictive models of breast cancer risk for the Brazilian population.

**Method:**

A cross-sectional, study was conducted in a sample of 382 women aged 35-69 years who were users of the Unified Health System (SUS) residing in a municipality in southern Brazil.

**Results:**

The results showed that the Tyrer-Cuzick model had the highest mean risk values and estimates (proportion) for predicting the 5-year risk of breast cancer, reaching a maximum risk of ±1.63% in the 60-64 year age group. For the 90-year risk, a maximum risk of ±12.8% was predicted for the 50-54 year age group using this model. The 5-year risk calculated by the three tools increased progressively with increasing age, where the mean risk was ±0.8% in women aged 35-39 and reached ±1.50% in women aged 65-69. The 90-year risk declined with increasing age only in the Tyrer-Cuzick model, from ±10.8% to ±9%. The BRCAPRO model presented a greater sensitivity compared to the Gail and Tyrer-Cuzick models. And, the model that presented greater specificity was Gail.

**Conclusion:**

The Tyrer-Cuzick model presented the highest risk estimates for 5 years and 90 years in the studied population, however, this data is not enough to validate this tool, since when analyzing the sensitivity and specificity the BRCAPRO and Gail have the highest values respectively.

## Introduction

Breast cancer is the most common type of cancer in women, accounting for more than 508,000 deaths worldwide (Global Health Estimates, WHO, 2013). Globally, a woman is diagnosed with breast cancer every 3 minutes, for a total of one million new cases per year (Babita et al., 2014). The incidence varies by region, with 27 cases per 100,000 women in Central and East Asia and 96 cases per 100,000 women in Western Europe (IARC, 2012).

In Europe, 28% of new cancer cases are breast cancer. In Brazil, the estimate for the 2018 – 2019 biennium is 59,700 new cases of breast cancer, with an estimated risk of 56.33 cases per 100,000 women. In the state of Paraná, Brazil, the estimated rate for 2018 was 64.70 cases per 100,000 women (INCA, 2017).

Although breast cancer is considered to have a good prognosis, there has been an increase in both its incidence and mortality, which may be associated with diagnoses performed at advanced stages (Gonzaga et al., 2014; Badan et al., 2014; OMS, 2018).

Screening is an important method of testing or examining an asymptomatic, apparently healthy population to identify lesions suggestive of cancer. It is considered a primary care technology and its indication may vary according to the target population, screening periodicity and method (Diretrizes, MS, 2017). Studies have shown that in countries with organized screening programmes, the mortality from breast cancer has been reduced (Myers et al., 2015; Feig, 2014). Thus, to organize the screening methods, several risk calculation models have been developed to predict a woman’s risk of developing breast cancer over the lifetime. Among these models, the most used are the Gail, BRCAPRO and Tyrer-Cuzick models (Reyes, 2009).

The developed risk calculation tools for rapid screening are easy to use and effective for breast cancer screening (Zhang et al., 2018). The predictions derived from the models are being used more frequently in the development of guidelines and recommendations for clinical care, in which women with a higher predicted risk are being advised to initiate mammography screening earlier and to consider magnetic resonance imaging (MRI) screening, genetic testing and chemoprevention (Coopey et al., 2018).

The American Cancer Society (ACS) and the National Comprehensive Cancer Network (NCCN) recommend MRI screening in women with a lifetime risk of breast cancer ≥20% as estimated by the Tyrer-Cuzick model, genetic testing in women with a risk for BRCA gene mutation of ≥10% calculated by the BRCAPRO model, and chemoprevention in women with a 5-year risk of breast cancer ≥1.67% calculated by the Gail model (NCCN, 2016; Visvanathan et al., 2013).

The tools for calculating risk as a screening method are important to classify the risk of the population so that screening methods specific to the population classified as high risk can be used for monitoring them, thus ensuring the early detection of breast cancer and therefore improving the disease prognosis. In this scenario, despite the validation of these screening models in several countries, their applicability differs in specific populations. The Tyrer-Cuzick and BRCAPRO models have not yet been validated in the Brazilian population; that is, there are still no studies evaluating their applicability in this specific population. Furthermore, studies on the Gail model only applied this model to populations who already had breast cancer, and thus cohort studies in the asymptomatic population evaluating the applicability of this model are lacking.

In view of the above, the objective of this study was to analyse the agreement between the Gail, Tyrer-Cuzick and BRCAPRO models regarding the obtained risks for breast cancer and to identify the best model for screening in the Brazilian population. 

## Materials and Methods

Cross-sectional study of a sample of 382 women aged 35 to 69 years. The female population in this age group residing in Paranavaí - Pr, according to IBGE data (2010), is 17,672. After the sample calculation based on the total number, with 5% error and 95% confidence level, a sample is 243 women plus 10% for possible tests, with a final sample of 267 women, who are users of the Unified System. (SUS) in the 13 primary care units of the municipality. Women who previously had or still had breast cancer were excluded.

Data were collected between March and September 2018 through a semi-structured instrument that was designed to explore the study objectives. The questionnaire contained 3 sections covering the main topics: (a) Characteristics of the study population such as socioeconomic and demographic factors, including age, educational level, city of residence, self-reported race or ethnicity, family income, and civil status; (b) Behavioural data, including weight, height, smoking, and alcohol consumption; and (c) Reproductive history and family history, including family history of breast cancer, age at menarche, number of children, breastfeeding, onset of menopause, and hormone replacement. The interviews were conducted by the researcher and by individuals trained by him. 

The data were tabulated in Microsoft Office Excel® 2010 spreadsheets, and the Rx64 software version 3.5.1 was used for statistical analysis. The risk calculations were performed with the CRA Health system (http://www.crahealth.com/), which uses the Gail, Tyrer-Cuzick and BRCAPRO models to calculate the 5-year and lifetime risks. The variables used in the calculation were the same in all tools. Women were considered to have a high lifetime risk for breast cancer if the predicted risk was ≥20% and were considered to have a high 5-year risk if the predicted risk was ≥1.67%.

The women were grouped by age into 5-year increments to compare the risks between the tools. The kappa coefficient was calculated to evaluate the level of agreement and reproducibility between the BRCAPRO and Gail, Tyrer-Cuzick and Gail, and BRCAPRO and Tyrer-Cuzick tools for the 5-year and lifetime risk. Analysis of variance (ANOVA) with a post hoc test was used test to the equality between the means of the tools, based on the analysis of the sample variances.

The analysis of sensitivity, specificity, positive predictive value and negative predictive value was performed per variable to detect which of the three tools is most appropriate for the study population. 

The sensitivity test has the diagnostic / screening ability to detect true positives, and the specificity, the diagnostic / screening ability to detect true negatives. While the sensitivity and specificity of a test are inherent properties of the test and do not vary except by technical error, positive predictive and negative predictive values depend on the prevalence of “disease” in the study population. Positive predictive value increases with disease prevalence while negative predictive value decreases (Reis and Reis, 2002). For this analysis was used the software R Core Team. R: A Language and Environment for Statistical Computing. Vienna, Austria: F Foundation for Statistical Computing; 2014.

To establish the cut-off point for the studied variables, used a variation of the ROC curve denominated TG-ROC (two-graph receiver operator characteristic), that is an alternative way of express graphically the relationship between sensitivity and specificity to help identify the best cut-off point. (Medronho, 2006). The sensitivity and specificity calculation was performed at the 10, 20, 30, 40, 50, 60, 70, 80, 90 and 100 percentiles to adopt the cutoff point.

The ethical aspects of the present study are based on the Guidelines and Norms Regulating Research involving Human Subjects (BRASIL, 2012b), according to Resolution 510/2016 of the National Health Council of Brazil (Conselho Nacional de Saúde). The study was approved under opinion number 2,251,844 of the Research Ethics Committee of the State University of Maringá (Universidade Estadual de Maringá).

## Results

Regarding the risk factors assessed by the three models in this study, which are age, age at menarche, age at first birth, history of cancer in first-degree relatives and race, the age ranged from 35 to 69 years, with an average age of 50 years. The age at menarche ranged from 7 to 22 years, with a mean of 12.8 years, and the age at first birth ranged from 11 to 42 years, with a mean of 22 years. Overall, 15.27% of the women were nulliparous, and 19.4% had at least one first-degree relative (mother, sister or daughter) with breast cancer. Regarding race, 40.15% of the women were of mixed race, 44.62% were white, 12.34% were black, 2.62% were Asian and 0.26% were indigenous.

**Figure 1:**
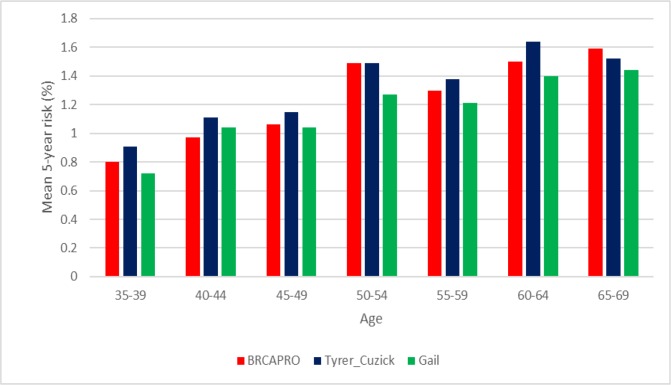
Mean 5-year Risk for Breast Cancer Calculated by the BRCAPRO, Tyrer-Cuzick and Gail Models in Brazilian Women

The mean 5-year risk for the development of breast cancer (Figure 1) was equal in the 50-54 year age group by the BRCAPRO and Tyrer-Cuzick tools, and in the other age groups the calculated mean was higher in the Tyrer-Cuzick tool, followed by BRCAPRO and Gail, except for the 40-44 year age group, in which the Gail model calculated a mean higher than the and in interval of the age between 65-69 years that the BRCAPRO model obtained a mean risk higher than Tyrer-Cuzick and Gail. Figure 1 also shows that the older the age group, the higher the calculated mean 5-year risk of breast cancer.

**Figure 2:**
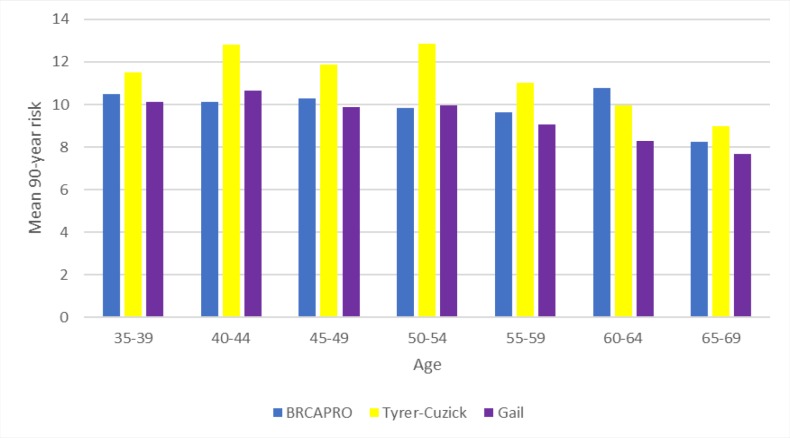
Mean 90-Year Risk for Breast Cancer Calculated by the BRCAPRO, Tyrer-Cuzick and Gail Models in Brazilian Women

According to Figure 2, the mean 90-year risks in the BRCAPRO and Tyrer-Cuzick models were higher in most age groups, except the 40-44- and 50-54-years groups, for which the means were higher in the Tyrer-Cuzick and Gail models. Thus, among the three tools, the Tyrer-Cuzick model calculated the highest mean for both the 5-year and 90-year (lifetime) risk. Additionally, the mean 90-year risk decreased with increasing age group for the three risk models.

According to the kappa analysis, the agreement between the tools was significant, with greater agreement between the Tyrer-Cuzick and Gail tools for both the 5-year and 90-year risk. The difference between the means was significant (p≤0.05) between the Tyrer-Cuzick and Gail models and between the BRCAPRO and Tyrer-Cuzick models for the 90-year risk (Table 1).

Regarding the 5-year risk, 37 cases of high risk were predicted with the Tyrer-Cuzick model, but these cases were predicted as low risk with the Gail model; the opposite occurred in 7 observations, where the Gail model resulted in a high risk and the Tyrer-Cuzick model in a low risk. Therefore, the Tyrer-Cuzick model presented greater sensitivity compared to the other tools.

When analysing the age-based risk proportion, the 5-year risk of breast cancer increased with increasing age group in all three tools (Table 2). The Tyrer-Cuzick model predicted the highest 5-year risks for breast cancer (≥ 1.67%) in most age groups. For the 90-year risk (≥20%), this model predicted the highest estimated risks across all age groups.

The 90-year risk of breast cancer decreased with increasing age according to the Tyrer-Cuzick model, but the same did not occur with the Gail model, as the risk remained high. The BRCAPRO model did not estimate any women as having high risk (Table 2).

The Table 3 shows the diagnostic accuracy of the study variables according to the three risk prediction models. The BRCAPRO tool calculated a higher mean of the sensitivity (46.4%) compared to the other tools, and the group of women receiving coverage under the Family Health Strategy showed the highest sensitivity (92.6%). The Gail model calculated the highest mean of the specificity (58.9%), but the group of women who underwent hormonal therapy, which presented higher specificity, was calculated by the BRCAPRO tool (97.3%), 

The Tyrer-Cuzick tool obtained higher Positive Predictive Value (PPV) for the group of women who never breastfed (34.1%) and also had the highest overall PPV, compared to the other models. The negative predictive value (NPV) was higher in the group of women who underwent screening exams for breast cancer (87.5%) and was calculated by the BRCAPRO tool, and the group of women who did not breastfeed (87.5%) was calculates by Gail tool. When analyzing the NPV in general, the Gail model presented a higher mean compared to the other models.

The cut-off point of variables analyzed as the woman’s age, body mass index and the woman’s age at the birth of the first child are presented in Figure 1, and for the woman’s age the 50 percentil equivalent to the age of 45 years was the same for the three tools. In the variable body mass index, the percentile was 50 = 26.4 (BMI) in the BRCAPRO tool, percentile was 45 = 27 (BMI) in the Tyrer-Cuzick tool and percentile was 40 = 25.4 (BMI) in the Gail tool. Already at the woman’s age at the birth of the first child the percentiles and cutoff points were the same in the three tools, percentile was 40 = 20 years.

**Table 1:** Level of Agreement between the Risk Tools, with kappa Coefficients. Brazil, 2018

Tools	n	Kappa index	p-value	Power of agreement	Adjusted p*
Gail and Tyrer-Cuzick– 5-year risk	381	0.631	0.000**	Substantial	0.983
Gail and BRCAPRO– 5-year risk	381	0.364	0.000**	Reasonable	0.999
BRCAPRO and Tyrer-Cuzick– 5-year risk	381	0.374	0.000**	Reasonable	0.999
Gail and Tyrer-Cuzick– 90-year risk	381	0.248	0.000**	Reasonable	0.000**
Gail and BRCAPRO– 90-year risk	381	0.000**	1.000	No agreement	0.392
BRCAPRO and Tyrer-Cuzick– 90-year risk	381	0.000**	1.000	No agreement	0.000**

* ANOVA with post hoc test; ** The adopted p-value was ≤0.05 (5% significance) for all calculated values.

**Table 2:** Proportion of Patients with 5-year Risk ≥1.67 and 90-year Risk ≥20, Based on Age. Brazil, 2018

Risk Model	35-39	40-44	45-49	50-54	55-59	60-64	65-69	Average
	(%)	(%)	(%)	(%)	(%)	(%)	(%)	(%)
Risk ≥1.67 - 5 years								
BRCAPRO	5.3	10.4	11.3	15.1	22.4	52.6	56.8	24.84
Tyrer-Cuzick	8.8	16.4	16.1	35.8	22.4	31.6	35.1	23.74
Gail	3.5	7.5	9.7	26.4	20.9	21.1	27.0	16.58
Risk ≥20 - 90 years								
BRCAPRO	-	-	-	-	-	-	-	-
Tyrer-Cuzick	1.8	11.9	8.1	11.3	4.5	2.6	-	6.7
Gail	1.8	3.0	1.6	1.9	-	2.6	-	2.2

**Table 3:** Analysis of Sensitivity, Specificity, Positive Predictive Value and Negative Predictive Value, Bsed on Variables. Brazil, 2018

Variable	Sensitivity			Specificity			VPP***			VPN****		
	BR Capro	TC**	Gail	BR Capro	TC	Gail	BR Capro	TC	Gail	BR Capro	TC	Gail
schooling <9 years	47	44.3	39.0	64.4	63.7	62.1	26.9	26.9	15.9	81.4	79.1	84.7
Race/color	55.4	51.1	52.5	44.3	43.2	43.8	21.7	21.3	14.6	78.1	74.6	83.4
Income <1MW	30.1	29.5	30.5	68.5	68.2	68.6	21.0	21.8	15.1	77.9	76.2	84.4
Coverage FHS	92.6	91.6	91.2	9.1	8.1	8.7	23.1	23.5	16.0	80.6	75.9	83.9
Visit FHS	86.6	79.1	82.5	15.2	12.9	14.2	23.2	22.3	15.4	79.2	66.0	81.1
Family history. BC*	10.8	12.5	9.3	77.9	78.1	83.0	12.0	14.7	11.9	75.8	74.8	78.9
Marital Status	47.0	44.3	47.5	61.1	60.6	60.6	25.2	25.3	18.1	80.5	78.3	86.3
Chronic disease	31.3	33.0	25.4	70.1	70.5	68.9	22.6	25.2	13.0	78.6	77.7	83.5
physical inactivity	63.4	70.1	69.5	31.9	33.6	33.3	20.4	23.9	16.1	76.0	79.0	85.6
smoking	9.6	10.2	13.6	87.6	88.0	88.5	17.8	20.5	17.8	77.7	76.5	84.8
alcoholism	23.2	21.8	22.4	77.5	77.4	77.3	22.1	22.4	15.1	78.6	76.9	84.7
performs tracking scan	86.7	77.3	74.6	25.8	23.3	22.7	24.6	23.3	15.0	87.5	77.3	83.0
did not breastfeed	84.5	18.9	25.5	12.0	89.0	89.4	21.5	34.1	29.3	73.2	78.5	87.5
hormonal therapy	4.8	3.4	5.1	97.3	96.9	97.2	33.3	25.0	25.0	78.5	76.8	84.8
contraceptive use	20.7	25.3	34.5	71.8	72.9	74.8	16.8	21.8	19.8	76.7	76.5	86.3
Do not know Ca Mama	48.2	50.0	42.4	50.3	49.3	49.4	21.3	22.9	13.3	77.7	76.6	82.4
Mean±standard deviation	46.4±29.7	41.4±26.9	41.6±26.3	54.1±28.0	58.5±27.4	58.9±27.6	22.1±4.6	23.4±4.0	17.0±4.5	78.6±3.1	76.3±3.0	84.1±2.1

* Family history of breast cancer; **Tyrer-Cuzick; ***Positive Predictive Value; ****Negative Predictive Value

**Figure 3:**
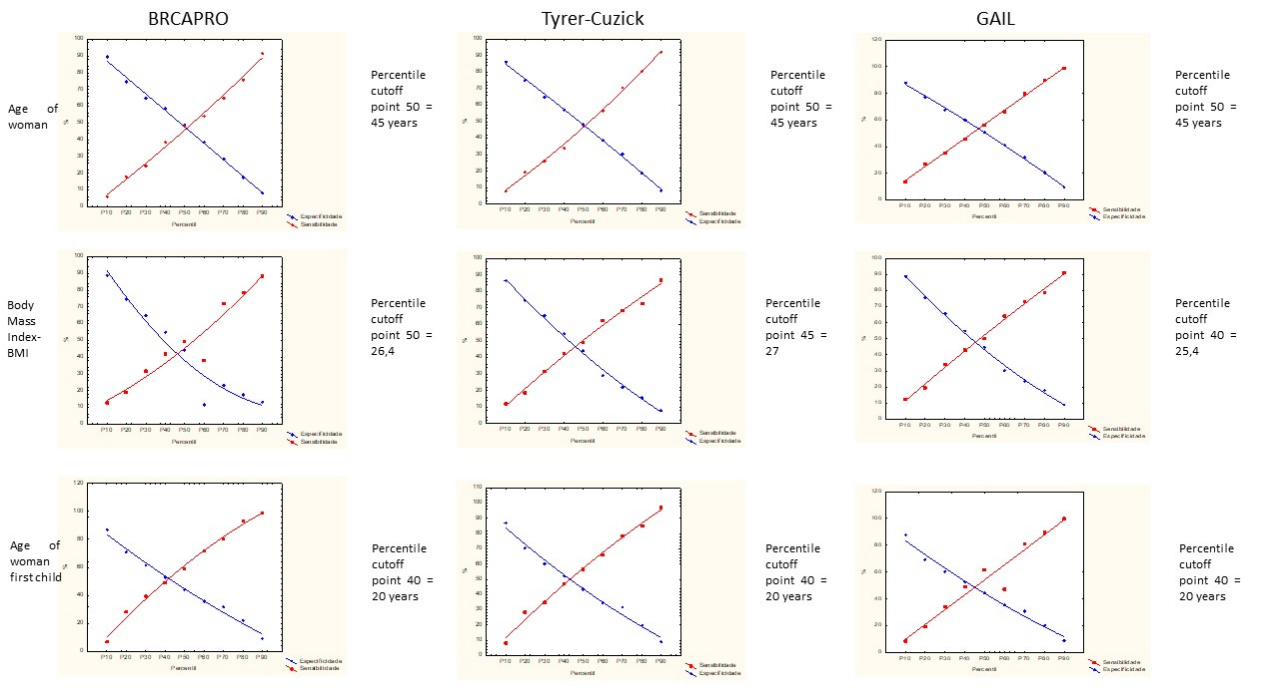
Cut-off Points for the Variables Woman's Age, Body Mass Index and Woman's Age in the First Child, According to the Three Risk Models, BRCAPRO, Tyrer-Cuzick and Gail

## Discussion

Tools for assessing risk as a screening method are important for classifying the population risk for breast cancer and for monitoring the population classified as high risk through other screening methods. Thus, models should be used that ensure the early detection of breast cancer in its initial stage, thereby improving the prognosis of the disease and the quality of life of women. Although validation of these screening models has been conducted in several countries, their applicability differs in specific populations. To date, despite the high rates of mortality due to breast cancer in the Brazilian population, there are still no correlation studies of these models and their effectiveness in screening for breast cancer. The Tyrer-Cuzick and BRCAPRO models have not yet been validated in the Brazilian population; there are no studies evaluating their applicability in this specific population. Studies on the Gail model have applied it only to populations already with the disease, and there are no cohort studies in an asymptomatic population evaluating the applicability of this model.

The estimated risk of breast cancer varied between the risk models. This is because each model uses a different statistical calculation for the same analysed variables. Additionally, each model has different risk factor variables for the statistical calculation. Thus, identifying the best risk model for the Brazilian population is important to provide specific recommendations according to the risk found and the age of the patient. According to the NCCN guidelines (2016), a risk equal to or greater than 20% calculated by a validated risk tool is an indication for MRI breast cancer screening. A risk equal to or greater than 1.67% is a criterion for the indication of chemoprevention.

The BRCAPRO model obtained the highest mean risk (24.84%) for calculating breast cancer risk in 5 years. For the 90-year risk calculation, the Tyrer-Cuzick tool had a higher mean risk (6.7%). In the study by Coopey et al., (2018), the Tyrer-Cuzick tool presented the highest estimates of risk for breast cancer throughout life in all age groups. In a study realized out by Weisstock et al. (2013), about risk assessment used the model of Gail and Tyrer-Cuzick, the mean overall risk was higher for the Gail tool (2.86%) than for Tyrer-Cuzick (2.63%).

When analyzing risk estimates by age group, the Tyrer-Cuzick model calculated the highest risk estimates for breast cancer in 5 years (≥ 1.67%), and for the risk in up to 90 years (≥ 20%) in most of the age groups and also presented the highest number of observations, according with analysis of kappa. Brentnall et al. (2015) also evaluated these models in the United Kingdom with a cohort study and observed a higher diagnostic value of the Tyrer-Cuzick model in comparison to the Gail model, reinforcing the results of the present study. Ewans et al., (2016) compared the Tyrer-Cuzick, Gail and Breast and Ovarian Analysis of Disease Incidence and Carrier Estimation Algorithm (BOADICEA) models and found greater risk accuracy for the Tyrer-Cuzick model in a cohort of 6,268 women in the United Kingdom.

The 5-year risk calculated for this population by the three tools increased progressively with the age of the women, and the 90-year risk decreased with age when using the Tyrer-Cuzick tool. Using these models, Coopey et al., (2018) found a similar result in the American population, where the calculated lifetime risk of breast cancer decreased with patient age.

This study also showed that the BRCAPRO tool presented a sensitivity in the studied sample with a mean of 46.4% among all variables analyzed. The specificity, positive predictive value and negative predictive value were higher in the Gail tool with the respective values 58.9%, 23.4% and 84.1%. These results diverge from the study by Zhang et al. (201.8), which showed that the Tyrer-Cuzick model presented higher sensitivity (66%), specificity (86.92%) and positive predictive value (85.34%) for breast cancer risks, compared to the Gail model with 53.33%, 77.69% and 73.39 % respectively. In another study, conducted in India by Challa et al., (2013) showed a sensitivity and specificity of the Gail model closer to the result of the present study, in which the sensitivity was 51.9% and the specificity 64%, however, concluded that from these data it is still not possible to validate this tool in the Indian population, thus requiring more studies with a larger population.

The Gail model is the most validated model worldwide, but in Brazil its applicability remains questionable, since the most recent studies of this tool did not use a retrospective cohort to evaluate its performance. In the study by Lopes et al., (2014), which evaluated 105 women with breast cancer in Minas Gerais, the 5-year risk of breast cancer was underestimated. In a case-control study with women from the Brazilian state of Bahia, the 5-year risk was underestimated in the control group compared to the case group (Cruzoé et al., 2015). However, the Gail model is considered to predict risk in asymptomatic women; thus, the applicability of the Gail model in the Brazilian population is questionable, since no cohort study has evaluated risk in this population after years of monitoring.

A limitation of this study is that it was performed in a small sample of the Brazilian population, and the variables used by the three tools were the same, thus reducing the accuracy of the risk prediction by tools that have more specific risk variables for the statistical calculation. However, when analysing the same variables, the tools performed differently. The BRCAPRO model demonstrated a greater sensitivity for the prediction of breast cancer risk in Brazilian women compared to the Gail and Tyrer-Cuzick models.

In Brazil, the use of these risk prediction tools remains limited due to the lack of knowledge of the existence of these tools by some professionals, the culture of professionals in regard to resisting new technologies and especially the reliability of the results obtained, since there is still no model that evaluates the specific risk factors of this population, and the applicability of the existing models has not yet been verified through cohort studies.

In conclusion, the Tyrer-Cuzick model presented the highest risk proportion for both 5 years and 90 years in the studied population. However, this data is not enough to validate this tool, since that when analized the sensitivity, specificity, PPV and NPV, the BRCAPRO and Gail models present the highest values, respectively. It was also concluded that when the diagnostic accuracy of these three tests were determined, they were not sensitive enough to detect risk when there was actually risk, but they showed a greater capacity to determine absence of risk when the result was negative. Therefore, future studies are necessary to evaluate the applicability of these models in this population and to determine which tool is most appropriate considering the risk factors of Brazilian women, or even the development of specific models for this population.

## References

[B1] Almeida LMN, Conceição GA (2013). The young woman’s knowledge about the prevention of breast cancer. Portuguese Rev Enferm UFPI.

[B2] Amir E, Evans DG, Shenton A (2003). Evaluation of breast cancer risk assessment packages in the family history evaluation and evaluation program. J Med Genet.

[B3] Babita R, Kumar N, Karwasra RK (2014). Reproductive risk factors associated with breast carcinoma in a tertiary care Hospital of North India: A case–control study. Indian J Cancer.

[B4] Badan GM, Roveda Junior D, Ferreira CAP (2014). Complete internal audit of a mammography service in a reference institution for breast imaging. Radiol Bras.

[B5] Barcenas CH, Hosain GM, Arun B (2006). Related articles, assessing the likelihood of Brca carriers in extended families. J Clin Oncol.

[B7] Brentnall AR, Harkness EF, Astley SM (2015). Mammographic density adds precision to the breast cancer risk models Tyrer-Cuzick and Gail in a prospective screening cohort in the Uk. Breast Cancer Res.

[B8] Challa VR, Swamyvelu K, Shetty N (2013). Assessment of the clinical utility of the Gail model in estimating the risk of breast cancer in women from the Indian population. Ecancer.

[B9] Coopey AB, Acar A, Griffin M (2017). The Impacto F patient age on breast cancer risk Prediction Models. Breast J.

[B12] Feig SA (2014). Screening mammography benefit controversies: Sorting the evidence. Radiol Clin North Am.

[B13] Gonzaga CM, Freitas Junior R, Souza MR (2014). Disparities in female breast cancer mortality rates between urban centers and rural areas of Brazil: Ecological Time-Series Study. Breast J.

[B15] Jame PA, Doherty r, Harris M (2006). Optmal selection of individuals for Brca mutation testing: A Comparison of Available. Methods.

[B16] Leal EM, Almeida LMN, Lima AGS (2014). Conhecimento E Prática do autoexame Da Mama Em Usuárias De Um Centro De Saúde. Rev Enferm UFPI.

[B17] Lopes FC, Franco TLB, Gradim CVC (2014). Mensuração dos fatores de risco de mulheres com câncer mamário através do Índice de Gail. Rev Enferm.

[B18] Medronho RA, Magnani MMF, Torres TZG, Pereira BP (2006). Epidemiologia: Probabilidade E Distribuições De Probabilidade. São Paulo: Editora Atheneu.

[B20] Myers ER, Moorman P, Gierisch JM (2015). Benefits and harms of breast cancer screening: A systematic review. JAMA.

[B25] Sadoughi F, Afshar HL, Olfatbakhsh A (2016). Application of canonical correlation analysis for detecting risk factors leading to recurrence of breast cancer. Iran Red Crescent Med J.

[B26] Visvanathan K, Hurley P, Bantug E (2013). Use of pharmacologic interventions for breast cancer risk reduction: American Society of Clinical Oncology Clinical Practice Guideline. J Clin Oncol.

[B27] Weisstock CR, Rajapakshe R, Bitgood C (2013). Assessing the breast cancer risk distribution for women undergoing screening in British Columbia. Cancer Prev Res.

[B29] Zhang L, Jie Z, Xu S (2018). Use of the receiver operational characteristic curve (Roc) for Tyrer-Cuzick and Gail at the breast cancer screening in Jiangxi Province, China. Med Sci Monit.

